# HIV-1 Capsid Core: A Bullet to the Heart of the Target Cell

**DOI:** 10.3389/fmicb.2021.652486

**Published:** 2021-04-01

**Authors:** Elenia Toccafondi, Daniela Lener, Matteo Negroni

**Affiliations:** CNRS, Architecture et Réactivité de l’ARN, UPR 9002, Université de Strasbourg, Strasbourg, France

**Keywords:** HIV-1, capsid, uncoating, reverse transcription, cellular cofactors, restriction factors, genetic fragility, nuclear transport

## Abstract

The first step of the intracellular phase of retroviral infection is the release of the viral capsid core in the cytoplasm. This structure contains the viral genetic material that will be reverse transcribed and integrated into the genome of infected cells. Up to recent times, the role of the capsid core was considered essentially to protect this genetic material during the earlier phases of this process. However, increasing evidence demonstrates that the permanence inside the cell of the capsid as an intact, or almost intact, structure is longer than thought. This suggests its involvement in more aspects of the infectious cycle than previously foreseen, particularly in the steps of viral genomic material translocation into the nucleus and in the phases preceding integration. During the trip across the infected cell, many host factors are brought to interact with the capsid, some possessing antiviral properties, others, serving as viral cofactors. All these interactions rely on the properties of the unique component of the capsid core, the capsid protein CA. Likely, the drawback of ensuring these multiple functions is the extreme genetic fragility that has been shown to characterize this protein. Here, we recapitulate the busy agenda of an HIV-1 capsid in the infectious process, in particular in the light of the most recent findings.

## Introduction

Retroviral infection begins with the fusion of the viral and cell membranes, carried out by the viral envelope proteins (Coffin et al., 1997). This causes the entry in the cytoplasm of the viral capsid core (also simply referred here as the core), a shell constituted by approximately 1,500 copies of the capsid protein CA. The capsid core contains the viral genomic RNA (gRNA) and protects it from cellular sensors of innate immunity and antiviral factors. The infectious cycle requires the reverse transcription of the gRNA to convert it into double-stranded DNA. The capsid core favors this step by providing a confined environment where the concentration of the viral components is high. At the moment of integration, though, the genetic material must have been released from the core, in order to interact with, and integrate into, the chromosomes. When and how the protective shell is dismantled is still not clear. According to the earliest models, disassembling of the core occurred soon after its entry into the cytoplasm (Bukrinsky et al., 1993; Miller et al., 1997; Fassati and Goff, 2001). This view has been challenged recently by an increasing number of observations that support the idea that capsid cores remain intact or almost intact, long after their entry into the cell, and even once in the nucleus (Burdick et al., 2020; Dharan et al., 2020; Selyutina et al., 2020b). This implies that the core constitutes a protective shell all along the trip from entry to almost the occurrence of integration. This review focuses on these aspects of viral infection: how and where the capsid core is dismantled in the light of the latest observations and which cellular factors, including those that control its stability, it comes across during its longer than expected presence in the newly infected cell.

## Structural Bases Determining the Stability of the Capsid Core

**FIGURE 1:**
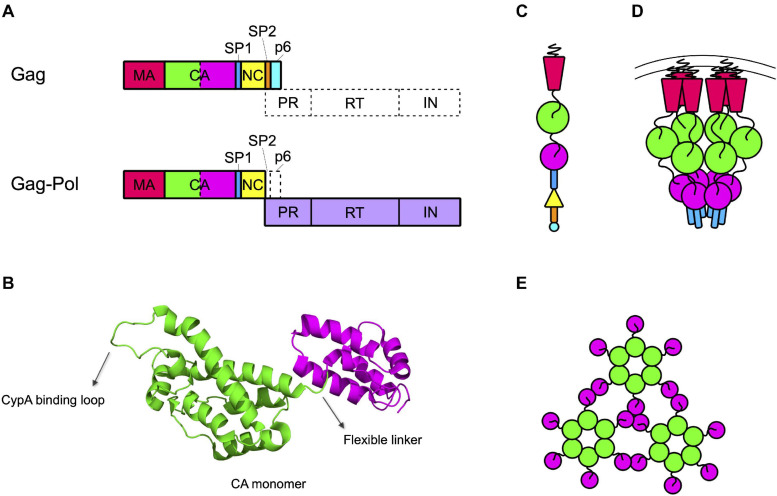
Capsid forms throughout the HIV life cycle. **(A)** Gag and Gag-Pol precursors simplified structures. Gag precursor includes the matrix protein (MA), the capsid (CA, depicted with the NTD in green and the CTD in magenta), the spacer peptide 1 (SP1), the nucleocapsid (NC), the spacer peptide 2 (SP2), and the peptide 6 (p6). A frameshift during translation allows the production of Gag-Pol precursor, with a ratio of 1:20 with respect to the Gag precursor. In this structure the NC is fused to the protease (PR), the reverse transcriptase (RT), and the integrase (IN) domains. **(B)** Structure of CA monomer. CA is composed of two domains connected by a flexible linker: the NTD (in green), formed by a beta-hairpin and seven alpha-helices, and the CTD (in magenta), formed by four alpha-helices. The CypA binding loop in the NTD is indicated. PDB ID: 6WAP (Lu et al., 2020). **(C)** Schematic structure of the Gag precursor composed from top to bottom of MA, CA-NTD, CA-CTD, SP1, NC, SP2, and p6. **(D)** Schematic structure of a hexamer in the immature lattice, after the first proteolytic cleavage, which occurs between SP1 and NC. The MA are attached to the membrane through their myristoylated domain. Proceeding toward the center of the viral particle there are three hexameric structures composed by the CA-NTDs, CA-CTDs, and SP1. **(E)** Schematic top view of the mature capsid lattice where CA monomers are arranged in hexamers and are connected to each other through the NTDs, while the CTDs are involved in the interactions between hexamers.

The capsid core is generated by the proteolytic processing of the Gag and Gag-Pol precursors that must free the CA protein. In the immature budding particle, these precursors assemble with each other to form the immature Gag lattice, a spherical protein shell located immediately underneath the lipidic envelope of the particle (Briggs et al., 2009). This structure is constituted by a vast majority of Gag precursors that include, from the N to the C terminus, the matrix (MA), the capsid (CA), the spacer peptide 1 (SP1), the nucleocapsid (NC), the spacer peptide 2 (SP2), and peptide 6 (p6) domains (Henderson et al., 1992; Figure 1A). Present in the lattice (at a ratio of approximately 1:20 with respect to Gag) are some molecules of Gag-Pol precursors, that contain MA, CA, SP1, and NC fused to the protease (PR), the reverse transcriptase (RT), and the integrase (IN) domains (Jacks et al., 1987; Reil et al., 1993; Figure 1A).

The structure of CA has been determined for the free protein, showing an organization in two globular domains (the N-terminal, NTD, and the C-terminal, CTD, domains) connected by a flexible linker (Figure 1B). The NTD is composed of seven alpha-helices and a beta-hairpin on the amino-terminal side while the CTD is composed of four alpha-helices (Gamble et al., 1996, 1997; Gitti et al., 1996). This structural arrangement has then been confirmed also for the CA domain in the Gag precursor (Tang et al., 2002; Schur et al., 2016; Wagner et al., 2016b). In the immature Gag lattice, MA points toward the exterior of the viral particle and, proceeding toward the interior, are present the NTD and CTD of CA and the SP1 domain, respectively (Figure 1C). Each of these domains multimerizes forming hexamers (Wright et al., 2007; Briggs et al., 2009; Schur et al., 2015, 2016). The interaction among CTDs of CA, stabilized by the six-helix bundles formed by SP1, is responsible for the formation of the immature Gag lattice, while the NTD of CA is not strictly required for assembly and it rather has the role of spacing the hexamers within the Gag lattice (Accola et al., 2000; Wright et al., 2007; Briggs et al., 2009; Bharat et al., 2012; Schur et al., 2016; Wagner et al., 2016b; Figure 1D).

**FIGURE 2:**
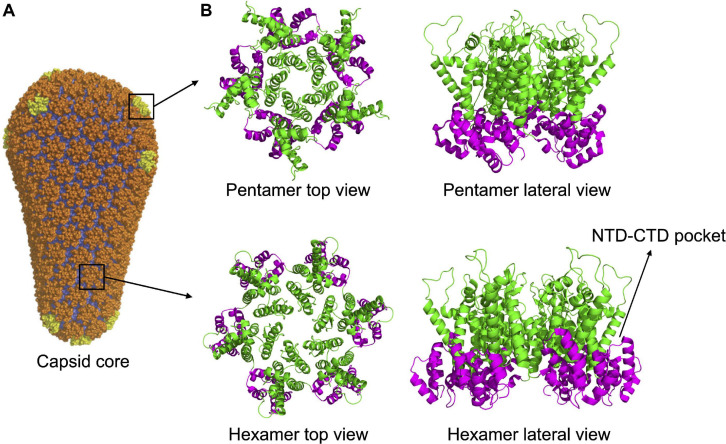
Capsid core structure. **(A)** The mature capsid core has the shape of a fullerene cone, formed by 125 hexamers (in orange) and 12 pentamers (in yellow). Image republished with permission of Nature Publishing Group (Pornillos et al., 2011). **(B)** Top and lateral view of pentameric and hexameric capsid assemblies. In both structures, the NTDs (in green) are forming the inner ring while the CTDs (in magenta) are forming the external ring. The pocket present in the hexamer, at the NTD-CTD interface (involved in the interaction with host factors, see main text) is indicated. The pocket is absent in the pentamer. PDB IDs: 5MCX, 5MCY (Mattei et al., 2016).

Multimerization, which occurs soon after budding, activates the viral protease, embedded in the Gag-Pol precursor. Once activated, the PR proceeds to an ordered sequence of cuts that cleave the Gag and Gag-Pol precursors into their individual components (Pettit et al., 1994, 2005). For CA, the first cleavage occurs at the junction between MA and CA. Subsequently, SP1 undergoes a conformational switch that allows the cleavage of the CA-SP1 junction releasing the free CA protein (Pettit et al., 2005). Once released, CA dissociates from the hexamers of the Gag lattice and spontaneously re-assemble to reform hexamers and form pentamers. The arrangement of CA NTD and CTD in the hexamers of mature capsid is different from that of the hexamers of the lattice. The orientation is inverted, with the NTDs that point toward the center of the structure and, by interacting with each other, stabilize the structure of the hexamer. The CTDs, in contrast, are located toward the exterior, in a radial disposition, and are involved in inter-hexamers interactions, holding together the capsid core (Ganser-Pornillos et al., 2007; Byeon et al., 2009; Pornillos et al., 2009; Zhao et al., 2013; Mattei et al., 2016; Figure 1E). Approximately 250 hexamers are involved, together with 12 pentamers, in the formation of the fullerene cone structure, 120 nm long and 60 nm wide (Ganser et al., 1999; Li et al., 2000; De Marco et al., 2010; Zhao et al., 2013; Figure 2A). Even for a given virus, the fullerene cones can vary in number of CA molecules, shape, and positioning of the 12 pentamers. This variability makes this structure highly pleiomorphic, which endows it with a certain conformational flexibility, an important feature for a viral component that has a central role in the interaction with several factors both of viral and of cellular origin (Ganser-Pornillos et al., 2004; Mattei et al., 2016). Pentamers are highly similar to hexamers in their structure, although the pocket between the CA domains in hexamers that, as discussed below, interacts with host factors, is unfolded in pentamers (Figure 2B). It is therefore expected that this interaction, if still occurring, is modified in the case of the pentamers. Also, the interactions between the monomers are slightly different in pentamers (Ganser et al., 1999; Cardone et al., 2009; Pornillos et al., 2011; Mattei et al., 2016). A detailed knowledge of the interactions established between CA monomers is important since several cellular components specifically recognize only the multimerized form of the protein, implying that the interactions between CA monomers generate functional elements *per se*.

## Turning Cell Proteins Into Viral Cofactors

The infectious cycle is strictly intertwined with the cell components. The viral proteins, indeed, interact with various cell proteins that can act as antiviral factors or as viral cofactors. Among these, some have been shown to interact directly with the capsid. They include the cyclophilin A (CypA) (Luban et al., 1993), the cleavage and polyadenylation specificity factor 6 (F6) (Lee et al., 2010), two proteins that are part of the nuclear pore complex (NPC) (Nup358 and Nup153), and the transportin 3 (TNPO3) (Brass et al., 2008; König et al., 2008; Table 1).

**TABLE 1:** Host factors interacting with the viral capsid.

Host factor	Gene	Biological role^a^	Role in HIV-1 Infection	Interaction with the capsid
Bicaudal D2 Protein	*BICD2*	Links the dynein motor complex to its cargos.	• Promotes the trafficking of viral cores toward the nucleus (Dharan et al., 2017).	Interacts with the assembled core through its C-terminal domain (Dharan et al., 2017; Carnes et al., 2018).
Cleavage and Polyadenylation Specificity Factor 6	*CPSF6*	One of the four subunits of the cleavage factor Im (CFIm), required for 3′-end RNA cleavage and polyadenylation processing.	• Participates in the nuclear import of the RTC/PIC complex (Chin et al., 2015; Burdick et al., 2020). • Involved in the choice of the integration sites (Chin et al., 2015; Rasheedi et al., 2016; Sowd et al., 2016; Achuthan et al., 2018; Francis and Melikyan, 2018; Bejarano et al., 2019).	Binds the hexameric form of CA in the nucleus at the NTD-CTD pocket (Lee et al., 2012; Price et al., 2012, 2014; Bhattacharya et al., 2014).
Cyclophilin A	*PPIA*	Cytoplasmatic peptidylprolyl *cis*-*trans* isomerase involved in proteins folding.	• Helps to maintain the stability of the capsid core (Li et al., 2009; Setiawan et al., 2016). • Involved in the choice of the nuclear import pathway (Schaller et al., 2011). • Protection from host restriction factors like TRIM5 (Kim et al., 2019; Selyutina et al., 2020a; Yu et al., 2020).	Binds to the capsid core in the cytoplasm by recognizing a conserved loop present in the NTD of CA (Franke et al., 1994; Gamble et al., 1996).
Extracellular Signal-Regulated Kinase 2	*MAPK1*	Serine/threonine-protein kinase part of the MAP kinase signal transduction pathway.	• Indirectly involved in promoting the uncoating step since its phosphorylation substrate is then recognized by Pin1 (Misumi et al., 2010; Dochi et al., 2014).	Phosphorylates the Ser16 of CA (Dochi et al., 2014).
Fasciculation and Elongation Protein Zeta 1	*FEZ1*	Kinesin-1 adaptor protein participating in the transport of cargos along microtubules.	• Promotes trafficking of the capsid core toward the nucleus (Malikov et al., 2015; Huang et al., 2019).	Binds the core at the hexamer pore (Huang et al., 2019).
Maternal Embryonic Leucine Zipper Kinase	*MELK*	Serine/threonine-protein kinase involved in many cellular pathways.	• Promotes viral uncoating (Takeuchi et al., 2017).	Phosphorylates the Ser149 of CA (Takeuchi et al., 2017).
MX Dynamin Like GTPase B	*MX2*	Interferon-induced dynamin-like GTPase protein located in the peripheric region of the nucleus.	• Blocks viral nuclear entry (Dicks et al., 2018; Kane et al., 2018). • Reduces integration efficiency (Kane et al., 2013; Liu et al., 2013; Matreyek et al., 2014).	Interacts with a negatively charged surface of CA (Smaga et al., 2019).
Non-POU Domain Containing Octamer Binding	*NONO*	RNA-binding protein with various roles in the nucleus including transcriptional regulation and RNA splicing.	• Restricts infection by activation of the immune response, *via* cGAS, after recognition of CA (Lahaye et al., 2018).	Binds to CA associated with the RTC/PIC complexes in the nucleus (Gao et al., 2013; Lahaye et al., 2013, 2018).
Nucleoporin 153	*NUP153*	NPC protein located in the nuclear basket of the complex with a role in the nucleocytoplasmic transport of proteins and mRNAs.	• Participates in the nuclear import of the viral complex (König et al., 2008; Matreyek and Engelman, 2011; Di Nunzio et al., 2012, 2013). • Directly or indirectly involved in the choice of the integration site (Koh et al., 2013; Marini et al., 2015).	It interacts with the multimeric form of CA at the NTD-CTD pocket at the same binding site of CPSF6 (Buffone et al., 2018; Bejarano et al., 2019).
Nucleoporin 358	*RANBP2*	RAN-binding protein located on the cytoplasmatic filaments of the NPC that promotes the nuclear import of large cargos.	• Favors the nuclear import of the viral complex (Schaller et al., 2011; Di Nunzio et al., 2012; Meehan et al., 2014; Dharan et al., 2016; Burdick et al., 2017). • Promotes uncoating of the capsid core at the NPC (Bichel et al., 2013).	Binds to the NTD domain of CA *via* a cyclophilin-homology domain as it approaches the NPC (Schaller et al., 2011).
Peptidylprolyl *Cis*/*Trans* Isomerase, NIMA-Interacting 1	*PIN1*	Peptidyl-prolyl *cis*/*trans* isomerase that specifically binds to phosphorylated ser/thr-pro motifs.	• Participates in the uncoating step (Misumi et al., 2010).	Recognizes the phosphorylated Ser16 of CA (Misumi et al., 2010).
Transportin 1	*TNPO1*	Involved in nuclear protein import as a receptor for nuclear localization signal.	• Involved in keeping the correct stability of the capsid core (Fernandez et al., 2019). • Helps the viral nuclear import (Fernandez et al., 2019).	Binds to the CypA binding-loop (Fernandez et al., 2019).
Transportin 3	*TNPO3*	Beta-karyopherin protein involved in the nuclear import of serine/arginine-rich (SR) proteins.	• Participates in the nuclear import step (Christ et al., 2008; Logue et al., 2011). • Involved in post-nuclear entry steps (Valle-Casuso et al., 2012; Shah et al., 2013). • Favors infection by participating in the nuclear localization of CPSF6 (De Iaco et al., 2013; Fricke et al., 2013).	Even if TNPO3 is also found in the cytoplasm, it most likely interacts with CA in the nucleus (Valle-Casuso et al., 2012; Shah et al., 2013).
Tripartite Motif Containing 5	*TRIM5*	Member of the tripartite protein family (TRIM) located in the cytoplasm of the cell where it autoassembles in cytoplasmic bodies.	• Affects the stability of the capsid core by either reducing it (Stremlau et al., 2006; Roa et al., 2012) or increasing it (Lu et al., 2015; Quinn et al., 2018). • Induces CA degradation *via* the proteasome (Lukic et al., 2011; Danielson et al., 2012; Kutluay et al., 2013) and/or the autophagy pathway (O’Connor et al., 2010; Mandell et al., 2014; Keown et al., 2018).	Forms a net around the intact capsid core in the cytoplasm by binding near or at the CypA binding site on CA (Quinn et al., 2018; Kim et al., 2019; Selyutina et al., 2020a; Yu et al., 2020).

*^a^Adapted from RefSeq.*

The first intracellular protein to be described to interact with HIV-1 CA was CypA that was identified through a two-hybrid screening of a human cDNA library of proteins interacting with Gag (Luban et al., 1993). Importantly, ever since this observation, the interaction with CypA has been shown not to be specific for HIV-1 but to be common among lentiviruses, for which it has been documented to exist for millions of years (Katzourakis et al., 2007; Gilbert et al., 2009; Goldstone et al., 2010; Malfavon-Borja et al., 2013; Mu et al., 2014). CypA is a peptidylprolyl isomerase that is incorporated in the viral particle *via* an interaction with G221 and P222 of Gag (G89 and P90 in mature capsid), and it is found with a stoichiometry of approximately 1:10 (CypA:Gag) (Franke et al., 1994; Braaten et al., 1996b). Despite the fact that CypA is packaged in the viral particle from the infected cell, which could suggest that it plays a role at the level of the producer cells, it has been shown that it is the interaction between CA and the CypA molecules present in the target cells to be the major determinant for the effect exerted by CypA on HIV-1 infection (Hatziioannou et al., 2005). CypA interacts with the capsid core in two different ways. On one hand, the active site interacts with G89 and P90 of the P_85_VHAGPIAP_93_ loop (Gamble et al., 1996; Figure 1B) and, due to its isomerase activity, could destabilize the core (Braaten et al., 1996a, b; Bosco et al., 2002; Ylinen et al., 2009). On the other hand, other parts of the protein contact the hexamer interface and, bridging hexamers, likely stabilize the capsid core (Liu et al., 2016; Ni et al., 2020). Indeed, the effect of CypA on infection is to alter the stability of the capsid core, albeit the results are rather controversial since, depending on the cell type, it has been shown either to increase or to decrease it (Li et al., 2009; Setiawan et al., 2016). However, since mutating the CypA binding site on CA or the use of cyclosporin A (CsA), a drug that competes with the CA for CypA binding, both severely interfere with HIV infectivity (Franke et al., 1994; Braaten et al., 1996b) it appears that the virus relies on the interaction with this cellular cofactor to reach the optimal stability of the core. Another role of CypA during infection is to avoid the recognition by the tripartite motif (TRIM) containing protein TRIM5 of the capsid core either by inducing a conformational change through its isomerase activity or by steric hindrance (Kim et al., 2019; Ni et al., 2020; Selyutina et al., 2020a; Yu et al., 2020). Finally, the interaction between CA and CypA also appears to regulate the pathway of nuclear import of the reverse transcription and/or pre-integration complexes (RTC/PIC) that differs, according to whether CypA interacts with CA or not (Schaller et al., 2011).

Many cytoplasmic factors interact with the capsid core, on its way to the nucleus. Bicaudal D2 protein (BICD2) and the fasciculation and elongation protein zeta 1 (FEZ1) are two dynein adaptor proteins, required for HIV-1 infection, that interact with HIV-1 assembled multimeric cores (Malikov et al., 2015; Dharan et al., 2017; Carnes et al., 2018; Huang et al., 2019). Their depletion results in impaired cytoplasmic trafficking, uncoating, and nuclear import (Dharan et al., 2017; Huang et al., 2019). Uncoating has also been shown to be influenced by other host factors, as Pin1, MELK, ERK2, and TRN-1. Pin1 is a peptidyl-prolyl isomerase that facilitates HIV-1 core disassembly by interacting with the phosphorylated Ser16-Thr17 motif (Misumi et al., 2010). Responsible for the phosphorylation of Ser16 is the extracellular signal-regulated kinase 2 (ERK2), a cellular factor that is incorporated in the viral particle through its interaction with CA (Dochi et al., 2014). Another kinase involved in destabilizing the viral capsid, in this case through phosphorylation of Ser149, is the maternal embryonic leucine zipper kinase (MELK). The mutant where Ser149 is replaced by the phosphor-mimetic amino acid Glu undergoes premature disassembly of the capsid core and is impaired in nuclear import of the reverse transcription products (Takeuchi et al., 2017). Finally, β-karyopherin transportin 1 (TRN-1) recognizes the CypA binding site with high affinity and it can displace CypA from its association to the core. Knock out of TRN-1 leads to reduced infection and premature uncoating (Fernandez et al., 2019). Overall, the trend observed with these factors indicates that they are required in order to maintain in balance the subtle equilibrium between uncoating and retention of a closed capsid required to accomplish infection. Defects in nuclear import observed by depleting these factors appear to be a consequence of alteration of capsid uncoating rather than a direct interference with the import process.

Nuclear pore complex proteins regulate trafficking between the nucleus and the cytoplasm in eukaryotic cells (Strambio-De-Castillia et al., 2010; Labokha and Fassati, 2013). Two of these proteins are well-characterized interactants of HIV-1 CA: Nup153 and Nup358 (also known as RANPB2) (Brass et al., 2008; König et al., 2008). Nup358 is associated with filaments that stem from the pore into the cytoplasm and it promotes the recruitment of nuclear import cargos (Hutten et al., 2009). It contains a cyclophilin-homology domain that is responsible for the interaction with CA (Schaller et al., 2011). As CypA, Nup358 has a *cis*-*trans* prolyl isomerization activity through which it can promote capsid core uncoating by catalyzing isomerization of CA (Bichel et al., 2013). This suggests that uncoating of the viral core could occur, at least partially, at the nuclear pore, once docked onto Nup358. Accordingly, depletion of Nup358 severely affects HIV-1 nuclear import, with a reduction of the amount of RTC/PIC docked at the NPC (Zhang et al., 2010; Schaller et al., 2011; Di Nunzio et al., 2012; Meehan et al., 2014; Dharan et al., 2016; Burdick et al., 2017). Nup153 is one of the components of the nuclear basket involved in the NPC formation and Nups recruitment (Vollmer et al., 2015). Through its C-terminal domain it binds the NTD-CTD pocket of CA (Buffone et al., 2018; Bejarano et al., 2019) and it favors its translocation into the nucleus (König et al., 2008; Matreyek and Engelman, 2011; Di Nunzio et al., 2012, 2013). Its depletion also alters the choice of the sites of integration (Koh et al., 2013; Marini et al., 2015). Since Nup153 binds CA hexamers with high affinity compared to monomeric CA (Matreyek and Engelman, 2011; Di Nunzio et al., 2012; Buffone et al., 2018), this translocation likely involves capsid cores that, if not intact, are at least partially assembled.

Another cellular protein interacting with CA is CPSF6, a pre-mRNA splicing factor, and a member of the serine/arginine-rich protein family (Rüegsegger et al., 1998). CPSF6 is part of the cleavage factor I (CFI_m_), together with CPSF5 and CPSF7, but its activities related to HIV-1 do not involve the other proteins of the complex (Rasheedi et al., 2016). CPSF6 binding site on CA is bipartite as CPSF6 binds at the N-terminal region of CA monomers but also at the NTD-CTD pocket of adjacent monomer on CA hexamers (Lee et al., 2012; Price et al., 2012, 2014; Bhattacharya et al., 2014). CPSF6 was initially identified to be relevant for HIV-1 infection through the functional screening of a mouse cDNA expression library that led to the isolation of a truncated form of CPSF6 (CPSF6-358) inhibiting HIV-1 replication (Lee et al., 2010). The truncation removes in CFSP6-358 the C-terminal arginine-serine like domain (RSLD) that is required for its nuclear import by transportin 3 (TNPO3) (Jang et al., 2019). As a consequence, the two forms of CPSF6 display different localizations inside the cell, with CPSF6 being predominantly nuclear while CPSF6-358 is found exclusively in the cytoplasm (Lee et al., 2010). This difference is responsible for the antiviral effect exerted exclusively by CPSF6-358 that blocks HIV-1 infection by interacting with the capsid core in the cytoplasm and preventing nuclear import (Lee et al., 2010). The integral form of CPSF6, in contrast, favors HIV-1 infection. Its effect is dependent on the cell type considered. Indeed, CPSF6 is an important factor in primary CD4+ T cells and macrophages, where it directs integration toward euchromatin regions, and its deletion leads to an accumulation of RTC/PIC complexes at the nuclear pore and integration in chromatin regions close to the nuclear pore (Chin et al., 2015; Rasheedi et al., 2016; Sowd et al., 2016; Achuthan et al., 2018; Francis and Melikyan, 2018; Bejarano et al., 2019; Burdick et al., 2020). These effects are not observed in HeLa or HEK 293T cells (Lee et al., 2010; Kane et al., 2018; Bejarano et al., 2019). The CPSF6 binding site on CA appears to overlap the region recognized by the nuclear pore protein Nup153, important for HIV-1 nuclear import, as discussed above, implying a competition for CA binding that could favor, once imported in the nucleus, the release from Nup153 to allow CPSF6 binding and its translocation into deeper nuclear regions (Bejarano et al., 2019).

Transportin 3 is a β-karyopherin that transports serine/arginine-rich splicing factors in the nucleus (Kataoka et al., 1999; Lai et al., 2000). It binds to HIV-1 CA and its depletion affects HIV-1 infection (Christ et al., 2008; Krishnan et al., 2010; Logue et al., 2011; Zhou et al., 2011; Valle-Casuso et al., 2012; Shah et al., 2013). The role of TNPO3 in HIV-1 infection is still debated. Some studies suggest a role in nuclear import (Christ et al., 2008; Logue et al., 2011) while others rather suggest an implication in post-nuclear import, but prior to integration (Zhou et al., 2011; Valle-Casuso et al., 2012; Shah et al., 2013). However, TNPO3 is also responsible for the nuclear import of CPSF6 (De Iaco et al., 2013; Maertens et al., 2014; Jang et al., 2019) which, in HIV-1 infection, favors nuclear transport, as discussed above. It is therefore possible that the effects on HIV-1 infectivity attributed to TNPO3 are not only direct but also a consequence of the effect of TNPO3 on CPSF6 (De Iaco et al., 2013; Fricke et al., 2013). In support of this view is the observation that another effect of the depletion of TNPO3 is a change in the choice of the integration sites (Ocwieja et al., 2011), which is the same phenotype observed when depleting CPSF6.

Besides assisting various steps of the infectious process from the mechanistic standpoint, as capsid uncoating or nuclear translocation, these host factors also have a role in the escape from innate immunity. For example, infection by viruses with mutated CA that no longer interact with several of these factors (CPSF6, CypA, and Nup358), triggers an interferon-mediated antiviral response in human monocyte-derived macrophages (Rasaiyaah et al., 2013). Consequently, the capsid is subject to positive selection for maintaining the interaction with these proteins. At the same time, it is also the target of several cellular factors endowed with antiviral activity, from which it has to escape, adding a layer of selective pressure. The most well-characterized of these factors are constituted by a member of the tripartite motif-containing proteins family TRIM5 (Stremlau et al., 2004), the myxovirus resistance gene A and B (MxA and MxB) (Liu et al., 2013), and the non-POU domain-containing octamer binding protein (NONO) (Lahaye et al., 2018; Table 1).

## Antiviral Factors Targeting the Capsid

An important cellular antiviral factor directed against the capsid is TRIM5α. TRIM5α was isolated from rhesus macaque (TRIM5α_rh_) in the context of studies aimed at understanding the reasons for the inability of HIV-1 to establish productive infections in Old World monkey cell lines (Shibata et al., 1995; Hofmann et al., 1999; Besnier et al., 2002; Cowan et al., 2002). Independently, a variant of this protein (TRIMCyp), exclusive to owl monkeys, was identified for its ability to confer the same phenotype of restriction to HIV-1 infection (Sayah et al., 2004; Stremlau et al., 2004). In both cases, the viral target was identified to be the capsid and, in particular, the assembled core rather than the monomeric form of CA (Cowan et al., 2002; Hatziioannou et al., 2004; Stremlau et al., 2006).

**FIGURE 3:**
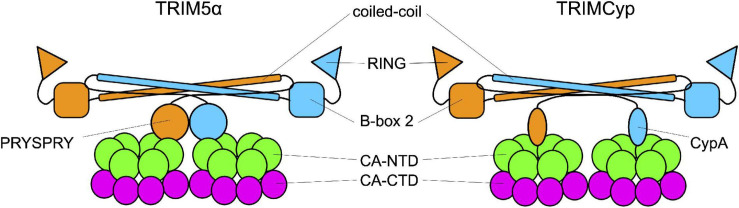
Interaction between TRIM and the capsid. TRIM5α and TRIMCyp are represented in their dimeric form. Each monomer (in orange and in blue) is formed by the RING domain, the B-Box 2 domain, the coiled-coil domain and the C-terminal domain which is the one responsible for the interaction with the capsid core. In TRIM5α this domain is the PRYSPRY domain while in TRIMCyp is CypA.

As members of the TRIM family, TRIM5α_rh_ and TRIMCyp are composed of a N-terminal tripartite motif constituted by the RING domain, a B-box 2 domain, and a coiled-coil domain (Reymond et al., 2001). The TRIM is followed by a C-terminal domain: cyclophilin A in TRIMCyp, and the PRYSPRY in TRIM5α. These domains bind the CA protein at or near the CypA-binding domain (Figure 3; Quinn et al., 2018; Kim et al., 2019; Selyutina et al., 2020a; Yu et al., 2020). TRIM5α and TRIMCyp dimerize through the coiled-coil domain, which places the two B-box 2 domains at each extremity of an antiparallel dimer. The B-box 2 domain can form trimers allowing the formation of a network of hexamers. These hexamers can assemble into a hexagonal lattice around an incoming retroviral capsid core, in which the C-terminal domains interact with the capsid (Sebastian and Luban, 2005; Li et al., 2016; Wagner et al., 2016a; Quinn et al., 2018; Yu et al., 2020). If the mechanisms of binding of TRIM5 to the capsid core are well understood, by which means it restricts HIV-1 infection is still debated. Some studies suggest that the ability of the protein to form a net around the capsid is sufficient to perturb the capsid core stability and, therefore, infectivity. The net would either induce the destabilization of the capsid core, resulting in a premature and non-productive uncoating (Stremlau et al., 2006; Zhao et al., 2011; Roa et al., 2012), or increase its stability by reducing the intrinsic flexibility of the core and of the CypA-binding loop in particular (Lu et al., 2015; Quinn et al., 2018). In both cases, infectivity would be perturbed. Other works indicate alternative pathways, activated by TRIM5α, to degrade the capsid core, as the recruitment of the proteasome, thanks to the ability of TRIM5α to undergo self-ubiquitylation, thanks to the RING domain (Fletcher et al., 2018) while associated to the capsid core (Lukic et al., 2011; Danielson et al., 2012; Kutluay et al., 2013) or by inducing selective autophagy of the capsid core (O’Connor et al., 2010; Mandell et al., 2014; Keown et al., 2018). However, neither blocking the proteasome nor the pathways leading to autophagy abolishes the restriction activity of TRIM5α suggesting that several, non-exclusive, pathways are activated in response to the recognition of the viral core (Perez-Caballero et al., 2005; Anderson et al., 2006; Diaz-Griffero et al., 2006; Wu et al., 2006; Kutluay et al., 2013; Imam et al., 2016; Keown et al., 2018).

The wealth of information concerning the restricting function of TRIM5α comes primarily from studies of the rhesus monkey protein. Indeed, the human ortholog of TRIM5α does not block HIV-1 infection in cell lines (Hatziioannou et al., 2004; Stremlau et al., 2004; Yap et al., 2004), although it protects human cells from the infection by some non-human retroviruses (Hatziioannou et al., 2004; Keckesova et al., 2004; Perron et al., 2004; Yap et al., 2004). Furthermore, a stabilized form of TRIM5α, obtained by producing a fusion protein with mCherry, protects human T cells in humanized murine models of HIV-1 infection (Richardson et al., 2014). Human TRIM5α is also involved in IFNa-induced inhibition against HIV-1 infection (Kane et al., 2016; OhAinle et al., 2018; Jimenez-Guardeño et al., 2019). In fact, high levels of IFNa activate the immunoproteasome, inducing a rapid turnover of TRIM5α that, being bound to the capsid core, drives to its degradation blocking viral replication (Jimenez-Guardeño et al., 2019).

The weak restriction of HIV-1 by human TRIM5α is suggested to be due to inefficient recognition of the capsid core (Stremlau et al., 2005; Yap et al., 2005; Merindol et al., 2018). The fact that the binding site of CypA on capsid cores overlaps (at least partially) the region bound by TRIM5α could lead to competitive inhibition of binding of TRIM5α, contributing to the inefficient recognition of the core by TRIM5α (Kim et al., 2019; Selyutina et al., 2020a; Yu et al., 2020). The lack of effectiveness of the human TRIM5α protein against infection with the human variant of the virus may reflect the recent exposure of humans to this virus. Alternatively, it could be imagined that HIV possesses a yet to be defined activity that counteracts that of TRIM5α.

The human *myxovirus resistance* (*Mx*) B protein (MxB, also known as Mx2) is an important anti-HIV factor that targets the viral capsid (Goujon et al., 2013; Kane et al., 2013; Liu et al., 2013; Matreyek et al., 2014). It is a dynamin-like GTPase, a family of proteins highly conserved in all vertebrates (Verhelst et al., 2013). MxB is constituted by a globular GTPase domain, a C-terminal stalk domain, a bundle signaling element (BPE), and a non-structured N-terminal domain (Gao et al., 2011). It localizes on the cytoplasmic side of the nuclear envelope, near the NPC (King et al., 2004). This antiviral factor is effective against herpesvirus, murine cytomegalovirus (MCMV), and HIV-1 (Goujon et al., 2013; Kane et al., 2013; Liu et al., 2013; Crameri et al., 2018; Jaguva Vasudevan et al., 2018; Schilling et al., 2018). In the N-terminal domain of MxB there is a positively charged motif, the ^11^RRR^13^ motif, that recognizes a negatively charged surface highly conserved among lentiviral capsid cores (Smaga et al., 2019). This interaction is responsible for the restriction of the infection (Goujon et al., 2015; Schulte et al., 2015) that, depending on the experimental conditions used, has been attributed either to a decrease of nuclear import of the RTC/PIC complexes by interfering with nuclear pore associated proteins (Dicks et al., 2018; Kane et al., 2018) or to a decrease of integration levels (Kane et al., 2013; Liu et al., 2013; Matreyek et al., 2014). Finally, a possible implication of MxB in the restriction response of the host restriction factor SAMHD1 has been recently suggested although it is still not clear how this is exerted (Buffone et al., 2019).

Another host factor with anti-HIV-1 activity related to targeting the viral capsid is the non-POU domain-containing octamer-binding protein (NONO), a member of the *Drosophila behavior/human splicing* (DBHS) family. The proteins of this family are characterized by the presence of two N-terminal RNA recognition motifs (RRMs), a NonA/paraspeckle domain (NOPS), and a C-terminal coiled-coil domain (Knott et al., 2016). NONO is a nuclear protein and has both RNA- and DNA-binding properties and it is involved in the activation of the innate immune response in dendritic cells and macrophages upon HIV infections, with a more efficient response against HIV-2 than HIV-1 (Lahaye et al., 2018). In the nucleus, NONO binds CA associated with the RTC/PIC complexes, and its restriction effect is exerted through the DNA sensor cyclic GMP-AMP synthase (cGAS), which activates the innate immune response by sensing the viral double-stranded DNA (Gao et al., 2013; Lahaye et al., 2013, 2018). Without NONO, cGAS is found in the cytosol and it does not activate the immune response (Lahaye et al., 2018).

## The Viral Uncoating Step and the Importance of Its Timing

The timing of dismantling of the viral capsid is a crucial aspect for a successful infection since premature disassembly would expose the components of the reverse transcription complex to the antiviral responses of the host cell and it would dilute the viral components by releasing them into the cytoplasm. On the other hand, the delayed dismantling of the capsid core could affect the process of integration by sequestering the reverse transcription products. To date, not only when and where reverse transcription and dismantling of the capsid core occurs is still an open question, but it is even still debated if and how the two processes are connected. Indeed, while some works show that DNA synthesis promotes uncoating (Hulme et al., 2011, 2015; Yang et al., 2013; Cosnefroy et al., 2016; Francis et al., 2016; Mamede et al., 2017; Rankovic et al., 2017), others show that the inhibition of reverse transcription neither affects uncoating nor the nuclear import of the RTC/PIC (Lukic et al., 2014; Burdick et al., 2017; Bejarano et al., 2019; Selyutina et al., 2020b).

Answering these questions is technically challenging, though. A major difficulty comes from the intrinsic properties of the capsid cores, discussed above, that is at the origin of the generation of polymorphic capsid cores, most of which intrinsically unstable and, therefore, non-infectious (Thomas et al., 2007; Mattei et al., 2016). It is, in fact, considered that only a minority of viral particles entering the cell leads to successful infection, while the majority is constituted by defective cores that undergo proteasomal degradation. The earliest studies on the capsid were mostly based on the biochemical tracking of the intact capsid in the infected cell. These analyses, consequently, followed the fate of the capsids at the “population” level and documented a rapid dismantling of the capsid after entry into the cell. The minority of stable capsids that, according to recent data, is responsible for productive infection, was not detected. The advent of techniques that allow following, by different means, the individual capsids has permitted focusing on the minority of capsids that persist in the cell changing our view of the timing of uncoating of the particles relevant for productive infection. The different scenarios that have been depicted for the dismantling of the capsid core are recapitulated hereafter.

### Cytoplasmatic Disassembly

According to the earliest models, uncoating occurs in the cytoplasm, soon after viral entry (early cytoplasmic disassembly) (Miller et al., 1997; Fassati and Goff, 2001). This model was supported by biochemical studies showing the lack of detectable CA in the cytoplasm (Bukrinsky et al., 1993; Miller et al., 1997; Fassati and Goff, 2001). However, increasing evidence showing the presence of CA and/or capsid cores in the cytoplasm of the infected cells has subsequently challenged this view (McDonald et al., 2002; Forshey et al., 2005; Shi and Aiken, 2006; Stremlau et al., 2006; Kutluay et al., 2013; Yang et al., 2013). It has thus been proposed that uncoating still occurs in the cytoplasm (Miller et al., 1997; Fassati and Goff, 2001) but delayed with respect to viral entry (late cytoplasmic disassembly) and coupled with reverse transcription (Hulme et al., 2011; Cosnefroy et al., 2016). A longer presence of an assembled capsid in the cytoplasm appeared also more plausible since it accounted for the protective role of the capsid from the exposure of the viral genome to host restriction factors and to the potential activation of the IFN-mediated antiviral response (Iwasaki, 2012). To date, it is accepted that uncoating in the cytoplasm concerns a fraction of the infecting particles and that, in general, it is only partial, with capsid hexamers that remain associated with the RTC/PIC complex, where they exert important functions in late steps of the infectious cycle (see below).

### Disassembly at the Nuclear Pore

As lentiviruses, unique among retroviruses, are able to infect non-replicating cells, entry into the nuclear compartment must proceed through the nuclear pore. Since the capsid core is larger than the nuclear pore, it was considered that the intact capsid could not be imported into the nucleus and, rather, it was blocked once docked at the level of the NPC (Arhel et al., 2007; Matreyek and Engelman, 2011; Schaller et al., 2011; Burdick et al., 2017; Francis and Melikyan, 2018; Francis et al., 2020; Zurnic Bönisch et al., 2020). Uncoating would then occur *in situ*, before import of the RTC/PIC could be possible. In support of this view came the measure of the time of residence of the viral complex at the nuclear pore that, for HIV-1, spans between 30 and 90 min (Burdick et al., 2017; Francis and Melikyan, 2018). Since macromolecular complexes of sizes similar to the RTC/PIC of HIV-1 have very short times of nuclear entry and a total binding time to the NPC of few milliseconds (Kelich et al., 2015), it was inferred that the longer time observed for HIV reflected the need for the capsid core to be dismantled and release the RTC/PIC. This way, the capsid core would protect the RTC/PIC from exposure to the proteasome until it has reached the proximity of the point of entry into the nucleus (Francis and Melikyan, 2018).

### Nuclear Disassembly

Increasing evidence, though, supports the possibility that, despite the apparent incompatibility in terms of size, the capsid core enters the nucleus intact or almost intact, and disassembles only once inside it. It has indeed been shown that several host factors interact, at the nuclear level, with the assembled capsid rather than CA monomers (Matreyek and Engelman, 2011; Di Nunzio et al., 2012; Valle-Casuso et al., 2012; Chin et al., 2015; Buffone et al., 2018; Bejarano et al., 2019). Furthermore, assuming that uncoating is favored by reverse transcription (Hulme et al., 2011, 2015; Cosnefroy et al., 2016; Francis et al., 2016; Mamede et al., 2017; Rankovic et al., 2017), if it constitutes a requirement for nuclear import of the RTC/PIC, blocking reverse transcription would be expected to affect nuclear import. This was not the case though, while increasing evidence supports a model where reverse transcription is completed only once in the nucleus (Burdick et al., 2017, 2020; Francis and Melikyan, 2018; Bejarano et al., 2019; Dharan et al., 2020; Francis et al., 2020; Rensen et al., 2020; Selyutina et al., 2020b). The most compelling evidence in favor of the idea that uncoating can occur in the nucleus then came from a series of recent works (Burdick et al., 2020; Dharan et al., 2020; Selyutina et al., 2020b). By labeling the capsid core with the GFP, producing a GFP-CA fusion protein, Burdick and coworkers observed that the core enters the nucleus while still intact (or almost intact), that reverse transcription is completed, and, finally, that uncoating occurs close to the integration sites approximately 1.5 h before integration (Burdick et al., 2020). In a concomitant work, Dharan et al. (2020) employed an inducible blockade of nuclear import at different time points and then evaluated the fate of the capsid cores that had entered the nucleus. In this setting, two main observations were made. One was that the completion of reverse transcription, as inferred by sensitivity to treatment with an inhibitor of reverse transcription, was posterior to nuclear import. The second observation was that, even after blocking nuclear import, the infection was susceptible to treatment with PF74. Since this compound inhibits infection through binding specifically the interface between CA monomers, these observations indicated that assembled (or partially assembled) capsid cores were present in the nuclear fraction. Finally, the observations that uncoating and reverse transcription are completed in the nucleus, have also been confirmed by the biochemical analyses of the purified cytosolic and nuclear fractions in infected cells by Selyutina et al. (2020b).

**FIGURE 4:**
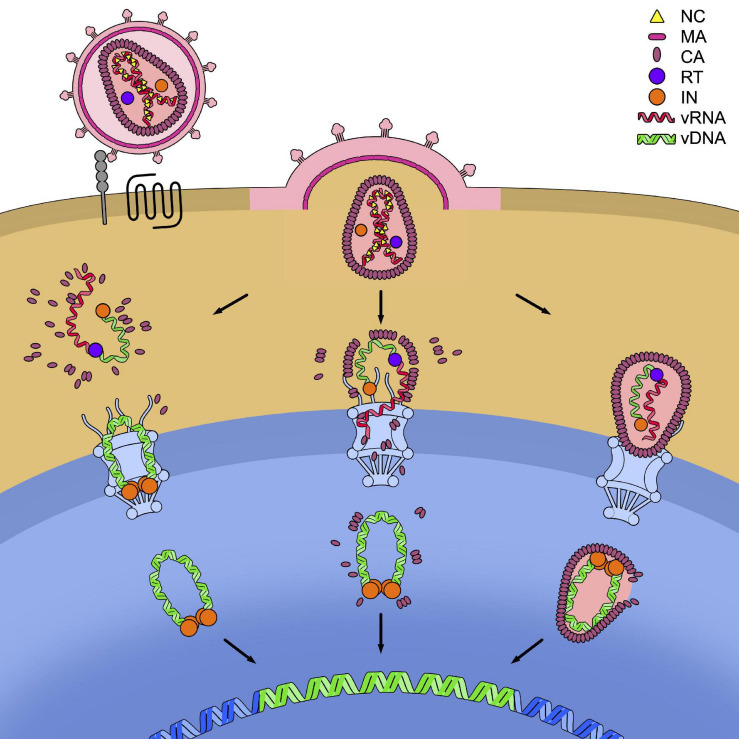
Models for the timing of uncoating. HIV-1 enters the cell after recognition by the envelope glycoproteins of the cellular receptor CD4 (in gray) and the cellular co-receptor CXC4 or CCR5 (in black). This leads to the fusion of the cell and viral membranes and to the release of the capsid core in the cytoplasm. In the figure, the three models of uncoating covered in this review are depicted: the cytoplasmic uncoating (on the left), the uncoating at the nuclear pore complex (NPC) (in the center), and the nuclear uncoating (on the right). In each model the reverse transcription of the viral genomic RNA (vRNA) (in red) into viral DNA (vDNA) (in green) has to be completed, allowing its integration in the host genome (in blue). The reverse transcription complex (RTC) is schematically shown as the association of a molecule of reverse transcriptase (RT, in purple) to the vRNA and single-stranded vDNA. The completed vDNA forms the pre-integration complex (PIC), shown as the double-stranded vDNA bound to a tetramer of integrase (IN, in orange).

These various models of dismantling of the capsid core are not mutually exclusive and it is possible that, depending on the cell type considered, the relative predominance of one or the other scenario is found. Might this be under the form of RTC/PIC deprived of CA, of a partially dismantled or of an intact capsid core, the viral element containing the genetic material must however, be translocated across the nuclear pore of the cell (Figure 4).

## Getting Into the Nucleus, Somehow

**FIGURE 5:**
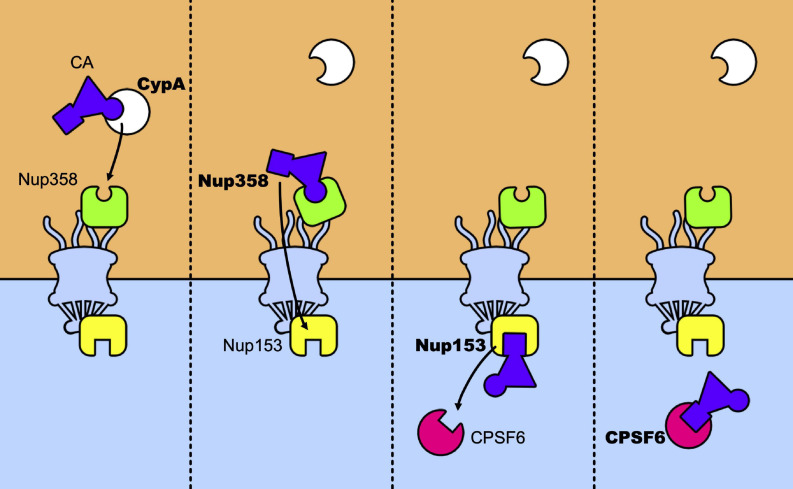
Relay race of the capsid core in the host cell. From left to right a temporal view of how CA is passed between host factors in its trip toward the nucleus. The capsid core is schematically represented as a purple triangle with two host factors binding sites highlighted: the CypA binding-loop (the circle) and the NTD-CTD pocket (the square). The first to bind to the core is CypA, which recognizes the CypA-binding domain, located in the CA-NTD. The same binding site is recognized by Nup358 and its binding anchors the capsid core at the NPC, allowing its nuclear import. Then, Nup153 binds to the NTD-CTD pocket of the assembled capsid, which is the same recognition site of CPSF6. When CPSF6 takes the place of Nup153 on the binding site it can translocate the capsid core (intact or not) to deeper nuclear regions.

The main nuclear import pathway of HIV-1 appears as a relay race where the capsid core is passed from CypA to Nup358, which passes it across the nuclear pore to Nup153 that will finally pass it to CPSF6 (Figure 5). However, alternative pathways exist. Mutants N74D and A77V of CA, identified for their less efficient binding to CPSF6 no longer require CypA, Nup153, Nup358, and TNPO3 (Lee et al., 2010; Schaller et al., 2011; Ambrose et al., 2012; Saito et al., 2016; Buffone et al., 2018). Despite this, they retain levels of infectivity comparable to those of the wt viruses, in primary cells. This suggests that, in these cells, alternative pathways are favored by these mutations. Concomitantly, these mutations induce uncoating at the nuclear pore and shift the integration sites to perinuclear regions (Burdick et al., 2020), in line with studies that show the importance of CPSF6 for nuclear import and the choice of the integration sites (Chin et al., 2015; Rasheedi et al., 2016; Sowd et al., 2016; Achuthan et al., 2018; Francis and Melikyan, 2018; Bejarano et al., 2019). Along the same lines, blocking transport across the nuclear pore by an inducible NPC blockade (Dharan et al., 2020), neither abolished nuclear import of the capsid nor blocked infection, indicating that nuclear pores can present a heterogeneous composition of nucleoporins and that factors alternative to the canonical Nup153, Nup358, and TNPO3 can also be used by the virus to achieve integration, in accordance with previous observations (Dicks et al., 2018; Kane et al., 2018). It is tempting to speculate that the use of these alternative factors is indicative of ancestral, less efficient, pathways at the expense of which the current canonical pathways of infection have evolved. On this note, the interaction between CA and CPSF6 seems to be preserved by selective pressure *in vivo* (Henning et al., 2014; Saito et al., 2016). This shift in the nuclear entry pathway would be a consequence of the use of previously unemployed cellular cofactors that allowed to optimize various steps of the infectious cycle and to improve escape from innate immunity.

Size also matters for nuclear import. Depending on where disassembly occurs (Figure 4), the nature and, consequently, the size of the complex that must cross the nuclear barrier changes considerably. The intact capsid core is around 60 nm wide (Briggs et al., 2003) while the nuclear pore is no larger than 40 nm (Von Appen et al., 2015). As discussed above this incongruence has long been considered a reason to exclude the possibility that the intact capsid core can be imported into the nucleus. Recently, by using a new method of visualization of capsid cores, based on immunogold labeling, Blanco-Rodriguez et al. (2020) showed that the capsid core undergoes important structural rearrangements before, during, and after nuclear import, leading to the formation of a pearl necklace-like shape that decorates the reverse transcribed DNA. The CA molecules, present in this structure that is considerably less wide than the intact capsid, could more easily mediate nuclear import. The possibility that structural rearrangements also involve the nuclear pore counterpart has been foreseen. Indeed, the NPC displays a marked structural flexibility that can be involved in the passage of large complexes as viral capsids (Knockenhauer and Schwartz, 2016; Mahamid et al., 2016). Furthermore, recent measurements of the inner diameter of the NPC by using cryo-EM on intact infected T cells have estimated a width of the internal channel of the pore of 64 nm, thereby slightly larger than the capsid core (Zila et al., 2021). The structure of the nuclear pore was dilated rather than rearranged with respect to previous observations made on HeLa cells where the canal appeared considerably narrower (Von Appen et al., 2015). In conclusion, increasing evidence supports the view that still “structured” capsid cores do enter the nucleus, this might be due to either partial uncoating that induces higher plasticity of the capsid core, either to structural rearrangements of the nuclear pore, either both.

## Genetic Fragility of the Capsid: A Mark of Multiple Constraints?

The retroviral capsid core is responsible for chaperoning the viral genetic material all along from the fusion of the viral and cellular membranes till its entry (or even after) into the nucleus. To accomplish this, the mature CA protein must meet several structural requirements to retain its ability to multimerize in order to assemble into the capsid core (and this relying on two different types of contacts, one giving rise to CA hexamers, the other generating pentamers, as discussed above), to interact with numerous cellular factors (Table 1) and to escape from adaptive immunity, being a target of cytotoxic T lymphocytes (CTLs) (Troyer et al., 2009). Furthermore, as a domain of Gag and Gag-Pol precursors, it must retain structural arrangements that do not interfere with the proteolytic processing of these molecules. Altogether, these constraints can account for the extreme genetic fragility of the protein (Rihn et al., 2013).

Genetic robustness is the ability to retain functionality when mutations are introduced in the protein (Visser et al., 2003; Wagner, 2005). Two main factors contribute to determining the genetic robustness of a protein. One is the number of functions the protein has to ensure and, consequently, the number of interactants it must come into contact with, in order to carry out its functions. The other is its architectural organization. For example, the presence of intrinsically disordered regions confers genetic robustness to proteins (Brown et al., 2002; Hultqvist et al., 2017). In the case of HIV-1 CA, the high number of partners it interacts with is likely the main determinant.

Local fluctuations in the degree of fragility are observed in CA. Internal regions of the protein are less tolerant of mutations as well as helices regions in the NTD rather than in the CTD and in the interhelical loops among which, surprisingly, the loop interacting with CypA. In particular, the region with the highest fragility is the one encoding the alpha-helices present in the NTD (Manocheewa et al., 2013; Rihn et al., 2013). This region is responsible, in the assembled core, for the interaction of each monomer with each other on the internal side of the hexamer, to form the internal ring (Figure 2B; Li et al., 2000; Pornillos et al., 2009, 2011). In addition, NTDs interact with the CTDs of adjacent monomers on the external portion of the CA (Lanman et al., 2003, 2004; Pornillos et al., 2009). These interactions must be finely tuned since during the extracellular life of the virus they must be stable enough to maintain a closed capsid core, but once inside the target cell they must allow the progressive dismantling of the structure, with the appropriate timing, as discussed above (Forshey et al., 2002). Maintaining this delicate equilibrium can account for the fragility of these regions. Of particular interest are the epitopes recognized by CTLs that appear particularly vulnerable to the introduction of genetic polymorphisms. A similar situation is found for the external regions of the HIV-1 envelope, which are the target of heavy artillery by the immune response, in this case humoral. It has been shown that in these regions the genomic sequence has evolved in such a way as to reduce the mutation rate (Geller et al., 2015), an observation interpreted as a mechanism to limit the cost of deleterious mutations, particularly high in these regions (Simon-Loriere et al., 2009; Hamoudi et al., 2013; Gasser et al., 2016). Marked genetic fragility could therefore constitute a common signature of regions under strong immune selection. Finally, several mutations that have a positive effect on viral replication *in vitro* were not found in natural populations, suggesting the existence of additional, presently unknown, sources of selection that counterselect some positive mutants but not others (Rihn et al., 2013). Identifying these sources of selection appears an important step for understanding the molecular bases of successful viral replication *in vivo*.

The marked genetic fragility of the capsid therefore likely derives from the cumulative requirements for interacting with a plethora of cellular factors that the virus has learned to deal with, for an optimal adaptation to its host. This fragility is probably responsible for the limited capacity of the capsid to avoid the immune response of the host (Troyer et al., 2009) and encourages to design new drugs targeting this protein. Drugs from which, in strict analogy to what occurs for the immune response, it should be difficult to escape.

## Concluding Remarks

The ultimate goal of a retrovirus is to reach the genetic material of the infected cell to integrate its own. To do so, the infectious cycle passes through two phases, an extracellular and an intracellular one. For each of these, a shell has been optimized. We now know that, as many vulnerable aspects of the envelope proteins are largely not accessible until the target cell has not been reached, also for the intracellular delivery of its genetic material, the virus does not leave a large window of opportunity for the host cell to sense and attack its genetic material. This, until the final destination is almost reached.

## Author Contributions

All authors listed have made a substantial, direct and intellectual contribution to the work, and approved it for publication.

## Conflict of Interest

The authors declare that the research was conducted in the absence of any commercial or financial relationships that could be construed as a potential conflict of interest.
